# Diffusive Propagation of Exciton-Polaritons through Thin Crystal Slabs

**DOI:** 10.1038/srep11474

**Published:** 2015-06-19

**Authors:** D. A. Zaitsev, N. D. Il’ynskaya, A. V. Koudinov, N. K. Poletaev, E. V. Nikitina, A. Yu. Egorov, A. V. Kavokin, R. P. Seisyan

**Affiliations:** 1 A.F. Ioffe Physico-Technical Institute, 26, Politechnicheskaya, 194021, St. Petersburg, Russia; 2 Spin Optics Laboratory, St. Petersburg State University, 1, Ulianovskaya, 198504, St. Petersburg, Russia; 3 St-Petersburg Academic University, 8/3, Khlopina, 194021, St. Petersburg, Russia; 4 ITMO University, Kronverkskiy pr., 49, 197101, St. Petersburg, Russia; 5 Physics and Astronomy, University of Southampton, Highfield, Southampton, SO171BJ, United Kingdom

## Abstract

If light beam propagates through matter containing point impurity centers, the amount of energy absorbed by the media is expected to be either independent of the impurity concentration *N* or proportional to *N,* corresponding to the intrinsic absorption or impurity absorption, respectively. Comparative studies of the resonant transmission of light in the vicinity of exciton resonances measured for 15 few-micron GaAs crystal slabs with different values of *N*, reveal a surprising tendency. While *N* spans almost five decimal orders of magnitude, the normalized spectrally-integrated absorption of light scales with the impurity concentration as *N*^1/6^. We show analytically that this dependence is a signature of the diffusive mechanism of propagation of exciton-polaritons in a semiconductor.

Exciton-polaritons are light-matter quasiparticles which may be excited by light in the spectral vicinity of exciton resonances in semiconductors^1^. They result from the quantum mechanical superposition of a massless photon state and a massive Wannier-Mott exciton state, which may be viewed as a chain of multiple virtual re-absorptions and re-emissions of photons. Exciton-polaritons (EPs) combine properties of excitons and photons, which makes them promising for applications in opto-electronics^2^,^3^ and leads to a great variety of non-linear effects including the Bose-Einstein condensation^4^, polariton lasing^5^, polarization switching^6^, dissipationless motion^7^, enhanced terahertz absorption^8^, etc. In spite of the great number of publications on real-space dynamics of exciton polaritons, their propagation, scattering and non-radiative decay in bulk crystal slabs still remain poorly understood (see, e.g., discussion in^9^). It is well known that the group velocity of an EP may vary from the speed of light in the semiconductor crystal down to the speed of a mechanical exciton which is several orders of magnitude lower, depending on the relative weight of excitonic and photonic fractions^10^. Due to their excitonic component, EPs efficiently interact with acoustic phonons, eventually decaying non-radiatively. Due to their photonic component, EPs can decay radiatively to vacuum photonic modes outside the sample^11^.

For these reasons, propagation of EPs in crystal slabs is governed by scattering events and an interplay of radiative and non-radiative decay processes. When inside the crystal, a polariton can only decay non-radiatively, while once it crosses the crystal slab and achieves one of the surfaces, it decays radiatively contributing to the reflectivity or transmission signal^12^. The spectrally resolved absorption of light in a crystal *A* is related to the reflectivity *R* and transmission *T* by the energy conservation law:


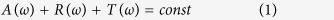


*A*(*ω*) accounts for all non-radiative losses including also the scattering to wave-guiding modes which decay through the side edges of the sample. What governs the energy dissipation in semiconductor crystals if light propagates in the spectral vicinity of an exciton resonance? What is the role of exciton-polaritons in the light absorption process? These questions are crucial for realization of polariton-based devices. They appear to be non-trivial both from experimental and theoretical points of view.

Direct measurement of the absorption is a hard task requiring the high accuracy measurements of the temperature variation of the sample frozen to a fraction of Kelvin^13^. Fortunately, one can obtain a good estimate of *A*(*ω*) from the spectral dependence of the transmitted intensity *T*(*ω*) through the Beer-Lambert-Bouguer law:


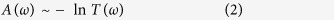


In Eq. (2) one assumes the exponential decay of the light intensity along its path inside the crystal slab (the Bouguer law). This assumption is generally valid in semiconductor films where the Perot-Fabry interference is suppressed because of the absorption, and the modulation of reflectivity in the vicinity of the exciton resonance is negligibly small^14^. More accurate extraction of the true absorption spectrum from the transmission spectrum can be done, e.g., using the transfer matrix method^15^.

In this paper, we present an experimental study of the absorption of light in doped semiconductor crystals in the EP regime, i.e., in the spectral vicinity of the excitonic optical transition. We have chosen GaAs crystals as the best-studied model semiconductor where optical phenomena and light-matter coupling have been studied for decades. We compare the spectrally-integrated excitonic absorption of light *K*, measured for 15 similar thin GaAs crystal slabs which differ by the concentrations of impurity centers *N*. While the excitonic absorption is an intrinsic effect and, at a first glance, should not depend on *N* at all, we have found a surprising sublinear dependence *K* ∝ *N*^*β*^ with *β* close to 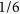. This observation sheds light on the mechanisms of non-radiative losses in semiconductors and allows concluding on the diffusive propagation of exciton-polaritons in doped semiconductors.

## Results

The samples for our studies were based on the epitaxial layers of GaAs, grown on the bulk semi-insulating GaAs substrates by means of either molecular beam epitaxy (MBE) or vapor phase epitaxy (VPE). The epitaxial layers were either nominally undoped or *p*-doped during the growth process. The impurity concentrations were then probed by either voltage–capacitance (VC) or Hall-effect measurements at room temperature. The measured values of the impurity concentration *N* are listed in Table 1 together with nominal dopants (for doped samples). The purest samples were not intentionally doped but contained residual impurities: most likely, S(As) which act as shallow donors with the binding energy of about 6 meV and the Bohr radius of about 90 Å. In doped p-type samples, the Si(As) atoms form acceptor centers with the binding energy of 34 meV and the Bohr radius of 13 Å.

After characterization, the substrates were removed by etching in ammonium and hydrogen peroxide water solution, and the epitaxial layers were etched down to sub-/few-micrometer thicknesses. The resulting thin crystal slabs were annealed in the atmosphere of hydrogen (in order to remove tension and oxides remaining on the surface after etching) and then loosely packed between two cover-glasses and sealed in the air. During optical measurements, these sandwiches were immersed in liquid helium at 2 K.

**Figure 1:**
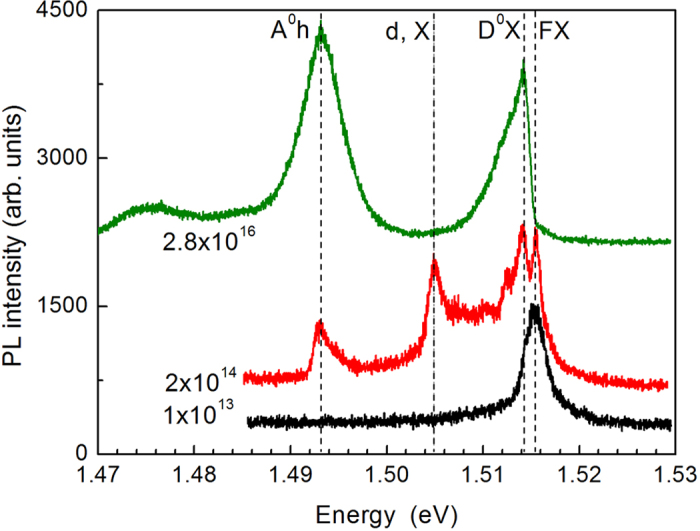
Representative PL spectra of sandwiched GaAs slabs: samples F235-B, R1488-1 and R1278-2. Excitation at 2.33 eV, T = 2 K. Assignment of the peaks as labeled.

As a first step, we measured the photoluminescence (PL) spectra for a selection of our samples in order to check possible effects of previous etching and sandwiching on the actual concentration of the impurity states. The PL was excited by the second harmonic of continuous-wave YAG:Nd laser (2.33 eV); the spectra were taken using a diffraction spectrometer and a photomultiplier. All in all, we have found that our slabs produce quite typical PL spectra of GaAs at the corresponding levels of doping. The observed features of the PL spectra can be readily assigned to various intrinsic or impurity-related optical transitions known from the optical spectra of GaAs, as shown in Fig. 1. Namely, the free exciton line (FX) at 1.5152 eV is easily identified by its well-known spectral position. Two lines represent excitonic complexes: the exciton bound to neutral donor (D^0^X) at 1.5142 eV possesses the binding energy ca. 1 meV, while the exciton bound to defect (d, X) at 1.505 eV, the binding energy of about 10 meV^16^,^17^,^18^,^19^. Finally, the (A^0^h) line at 1.493 eV should be ascribed to the *c*-band-to-acceptor optical transitions.

Remarkably, the PL spectrum of the slab with nominal *N* = 1⋅10^13^ cm^–3^ shows a single line of the free-exciton transition; the impurity-related features are not seen. This observation, first, confirms a very high purity of the F235 sample and, second, suggests that previous treatment did not really change the impurity concentration in our slabs. At the same time, the presence of the acceptor-related and donor-related lines simultaneously in one spectrum, as observed for some specimens (see upper two spectra in Fig. 1), points to a degree of compensation.

As a second step, we measured the transmission spectra of the samples. The “white light” from the incandescent lamp was first passed through the red filter which cut off the high-energy photons ( >1.9 eV), then focused on and passed through the sample, and finally focused on the entrance slit of a diffraction spectrometer. The pump density was about 1 W/cm^2^. The raw data on the spectrally-resolved transmission *I*(*ω*) for every sample were then normalized by the spectrum of the lamp. To this end, we had taken the reference spectrum *I*_0_(*ω*) of the lamp “white light” transmitted through the sample-box containing no sample inside.

**Figure 2:**
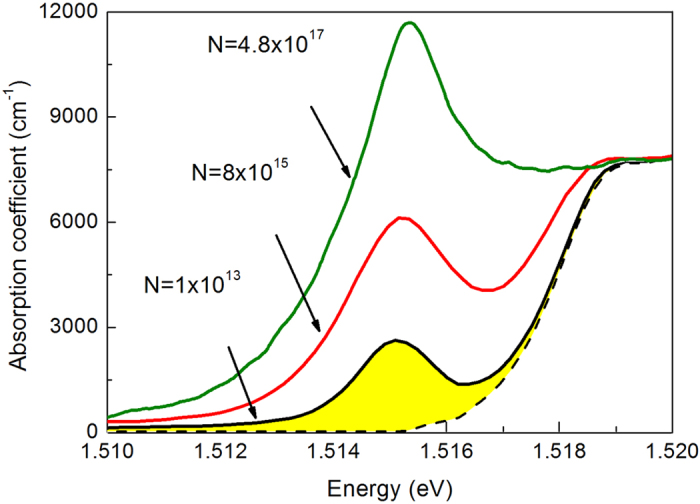
Representative spectra of the absorption coefficient in the vicinity of the fundamental absorption edge: samples F235-A, F-247-B, R1275-1. The spectra (solid lines) were extracted from the light transmission data as described in the text. For the lowest spectrum, decomposition into the excitonic contour (shaded area) and the non-excitonic absorption background (dashed line) is shown. The excitonic contour was obtained by mirror-reflecting the left half-contour, up to the maximum at ~1.515, to the right. The background was obtained (for the sake of visual control) by subtracting the excitonic contour from the bare experimental spectrum. Indeed, the background looks like a fundamental absorption edge accompanied by an Urbach tail.

At the next stage, the transmission data were processed further. We found the logarithm of the normalized transmission *I*(*ω*)/*I*_0_(*ω*) to obtain, having reversed the sign, the spectrum of optical density 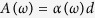. We aim at extracting the absorption coefficient *α*(*ω*) which should be an immanent characteristic of a corresponding semiconductor; however, the direct calculation is hindered because the slab thickness *d* is not accurately known and, moreover, is not quite homogeneous widhwise. Thus we took a straightforward assumption that the high-energy (interband) absorption coefficient should have the same value for any doping level, and we scaled the experimental optical density spectra of every sample in such a way that the high-energy shelves match the textbook value, about 8000 cm^–1^ for the bulk GaAs^20^ (see Fig. 2).

The absorption spectra shown in Fig. 2 demonstrate a distinct excitonic line peaked slightly above 1.515 eV, in the vicinity of the fundamental absorption edge for the interband transitions. While the peak energy is similar for all the samples, spectral widths and heights of the excitonic line vary for different samples. Thus, an integral value like the area under the excitonic absorption contour *K* = ∫*A*(*ω*)*dω* should be the most adequate characteristic of the efficiency of the excitonic absorption in a particular sample. One can clearly see the enhancement of the integrated exciton absorption with the increasing impurity concentration *N* (from lower to higher spectra in Fig. 2).

**Figure 3:**
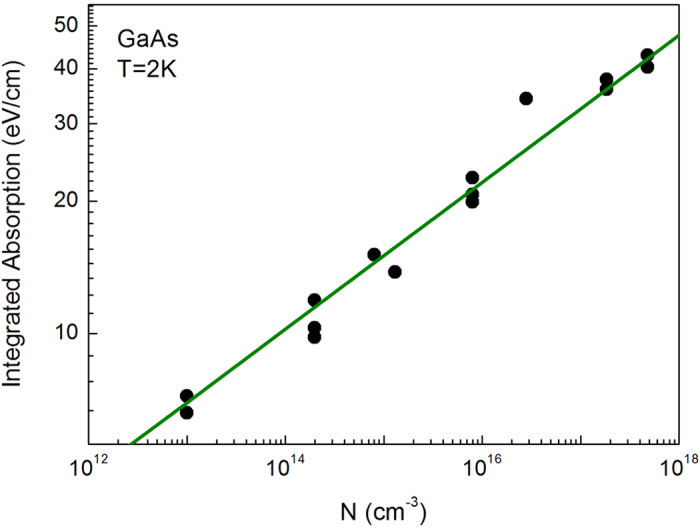
Energy-integrated excitonic absorption K versus the impurity concentration. Each experimental point represents a separate sample (see Table 1). Straight line shows the prediction of a diffusive model formulated here (Eq. 11).

For a quantitative analysis, the experimental spectra in Fig. 2 should be decomposed into the excitonic and non-excitonic contributions. The latter can include not only the classical fundamental absorption edge (whose spectral dependence might be known well enough), but the Urbach density-of-states tails as well, e.g., due to the Franz-Keldysh effect or disorder effects (whose spectral dependence is individual and is not precisely known). So the non-excitonic background can’t be merely subtracted, and the decomposition procedure needs a clear strategy relying on a healthy physical reasoning. The methods of analyses of the shape of the exciton absorption peak are discussed elsewhere^21^,^22^. In this study, we adapted a simple assumption that the low-energy part of every spectrum in Fig. 2, up to the energy of the excitonic peak, is not affected by the non-excitonic absorption. Thus we integrated under these half-bell-shaped parts (up to the maximum position), multiplied the result by a factor of 2 and consider as the full spectrally-integrated excitonic absorption, *K*. The obtained values of *K* are plotted in Fig. 3 against the corresponding impurity concentrations *N*.

Figure 3 summarizes the data on the variation of the integrated absorption with the impurity concentration. In the set of studied samples, *N* varies in wide limits, from *N* = 1⋅10^13^ cm^–3^ to about *N* = 5⋅10^17^ cm^–3^. For instance, in terms of electro-physical properties, such a range spans from semi-insulating to semi-metallic behaviour of bulk GaAs. But throughout the entire range, the dependence *K*(*N*) obeys a single power law, which corresponds to the straight line in a double logarithmic scale. A purely phenomenological two-parametric fit of the experimental trend by the function


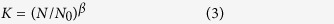


resulted in *β* = 0.175 ± 0.005, *N*_0_ = 10^8.3±0.9^cm^−3^. We shall see below that simple theoretical considerations give the value of the factor *β* = 1/6 ≈ 0.167.

## Discussion

The observed dependence of the integrated absorption on the impurity concentration is somewhat unexpected. Indeed, any kind of “impurity absorption” would be most naturally expected to demonstrate a linear dependence on *N*. A similar dependence of the integrated absorption on *N* was predicted for EPs by Akhmediev^23^ and reproduced in the recent theoretical work^24^. On the other hand, the excitonic absorption is an intrinsic phenomenon which might have been simply independent on *N*. These naïve expectations implicitly rely on the ideas of a ballistic propagation of photons through a matter where they rarely experience acts of inelastic scattering thus transferring photon energy to the crystal lattice. In contrast to that, in the presence of strong elastic scattering, the effective trajectory of each exciton-polariton propagating through the crystal may get significantly longer, which leads to the “slow light” phenomenon^25^. The scattering of exciton-polaritons by charged impurity centers and free carriers^26^,^27^,^28^ are expected to induce switching from the ballistic regime to the diffusive regime of polariton propagation. In what follows we show that in the diffusive propagation regime, the integrated absorption is governed by the characteristic time spent by diffusing polaritons in the crystal slab, which in turn depends non-linearly on the impurity concentration.

The propagation of EPs in a semiconductor slab containing a sufficiently high concentration of the diffusive centers can be described by a classical diffusion equation:


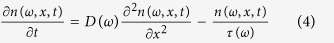


where *n*(*ω*, *x*, *t*) is the EP density, *D*(*ω*) is the frequency dependent diffusion coefficient, *τ*(*ω*) is the EP non-radiative lifetime which accounts for the absorption of light, *x* stands for the coordinate in the direction across the slab.

The diffusive propagation time of an EP through the crystal slab of the thickness *L* can be found from Eq. (4) according to Ref. [25]


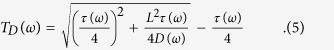


If the EP non-radiative lifetime is short enough (the strong absorption case), so that


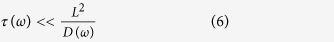


Eq. (5) reduces to


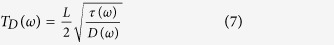


The diffusion coefficient is dependent on the mean free path of the exciton polariton *l*(*ω*) as


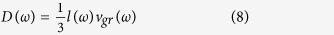


Here *v*_*gr*_(*ω*) is the EP group velocity which is defined by the dispersion of EPs in a bulk GaAs crystal, which is well-known and independent on the impurity concentration. In contrast, *l*(*ω*) is dependent on the impurity concentration. A mean free path of a plane wave packet is proportional to the average distance between the scattering centers, so that


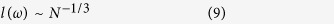


This relation is characteristic for EPs which are light waves with a specific non-linear dispersion and a coherence length frequently amounting to several μm^29^, while the average distance between impurity centers is of the order or less than 10 nm (see Table 1). The probability to find an impurity at the wave front of the plane wave is inversely proportional to the mean distance between impurities, which yields the relation (9). The proportionality (9) can be formally derived in many ways, but the simplest argument proving its validity is the dimensionality argument. If the lateral coherence length of propagating EPs is much larger than any characteristic length of the scattering problem, it can be assumed infinite. The relation between two remaining characteristic lengths, which are the mean free path and the mean distance between impurities, can be nothing but proportionality in this case, that readily yields the functional dependence (9).

Substitution of (9) into Eq. (7) yields


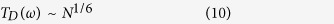


Now, the integrated absorption is governed by the time spent by light in the crystal slab, so that it can be estimated as


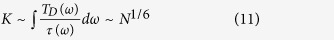


One can see that the diffusion model accurately reproduces the experimentally found functional dependence of the integrated absorption on the concentration of impurity centers in a semiconductor. This shows that, in the presence of impurities, propagation of EPs has a diffusive character. Unlike photons at neighboring energies (higher and lower), EPs travel through the slab along a polygonal line rather than ballistically. The absorption of ballistic photons is nearly independent of the impurity concentration, as can be seen from Fig. 2. At the low energy end (1.510 eV) it tends to zero, while at the high energy end (1.520 eV) it saturates at the value defined by the interband absorption.

Note that if EPs were classical particles whose coherence length is much less than the mean distance between impurities (and who would thus scatter on an isolated impurity center every time), the mean free path would depend on the impurity concentration in a different way: *l*(*ω*)~*N*^−1^ (cf. Eq. 9). This relation would yield, instead of Eq. (11), 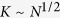, in contradiction with the experimentally observed trend.

Clearly, exciton-polaritons behave rather like extended wave-packets than like classical particles when propagating through a crystal with a sufficiently high impurity concentration. The strong absorption limit (6) applies only to relatively slow exciton-polaritons which are characterized by a high exciton component. These polaritons spend longer time inside the crystal slab and have better chances to decay non-radiatively than fast photon-like polaritons. They most essentially contribute to the integrated absorption *K*.

## Conclusions

In conclusion, the proof of diffusive propagation of exciton polaritons in semiconductor slabs has been obtained by a systematic study of the integrated absorption in a series of 15 GaAs samples. The dependence of the integrated absorption on the concentration of impurity centers unambiguously shows that the absorption of light is mostly through slow exciton-like exciton-polaritons which experience multiple scattering acts while propagating across the slab. This observation is important for understanding of mechanisms of the energy transfer between light and matter in semiconductors. The analytical model developed here works remarkably well at the liquid helium temperature. We expect that at higher temperatures the inelastic scattering processes would move a part of excitons outside the light cone, which may strongly affect the integrated absorption behavior.

## Additional Information

**How to cite this article**: Zaitsev, D.A. *et al*. Diffusive Propagation of Exciton-Polaritons through Thin Crystal Slabs. *Sci. Rep*. **5**, 11474; doi: 10.1038/srep11474 (2015).

## Figures and Tables

**Table 1:** Parameters of the samples used in this study.

**Sample Label**	**Growth Method**	**Impurity concentration, N (cm**^**−3**^)	**N measured using**
F235-A,B	VPE	1·10^13^	VC
R1488-1,3,4	MBE	2·10^14^	VC
F262-A	VPE	8·10^14^	VC
F244-C	VPE	1.3·10^15^	Hall&VC
F247-A,B,D	VPE	8·10^15^	Hall
R1278-1,2	MBE	2.8·10^16^ (Si)	Hall
R1144-2	MBE	1.85·10^17^ (Si)	Hall
R1275-1,3	MBE	4.8·10^17^ (Si)	Hall
